# Optimized approaches to profile low biomass DNA and RNA virome in the upper respiratory tract of beef cattle

**DOI:** 10.1186/s42523-026-00567-4

**Published:** 2026-10-13

**Authors:** Yue Wang, Mi Zhou, Nilusha Malmuthuge, Yanhong Chen, Trevor W. Alexander, Edouard Timsit, Le Luo Guan

**Affiliations:** 1 Faculty of Food and Land Systems, The University of British Columbia, Vancouver, BC V6T 1Z4 Canada; 2 College of Animal Science and Technology, Northwest A&F University, Yangling, Shaanxi China; 3 Department of Agriculture, Food and Nutritional Science, University of Alberta, Edmonton, AB T6G 2P5 Canada; 4 Faculty of Veterinary Medicine, University of Calgary, Calgary, AB T2N 1N4 Canada; 5 Lacombe Research and Development Center, Agriculture and Agri-Food Canada, 6000 C and E Trail, Lacombe, AB T4L 1W1 Canada; 6 Lethbridge Research and Development Center, Agriculture and Agri-Food Canada, Lethbridge, AB T1J 4B1 Canada; 7 Ceva Santé Animale, Libourne, 33500 France

**Keywords:** Cattle upper respiratory tract, Virome profiling, Optimization

## Abstract

**Background:**

Viruses play a key role in cattle respiratory tract (RT) health, yet, profiling the upper respiratory tract (URT) virome (DNA and RNA viruses) from deep nasopharyngeal swabs (DNS) remains challenging due to their low biomass, lack of well-defined enrichment approaches, and a high proportion of unclassified viral sequences. This study aimed to optimize DNS preservation, nucleic acid extraction, and bioinformatics pipelines to enhance URT virome characterization in beef calves.

**Results:**

Three DNS preservation methods were tested (RNAlater, brain heart infusion (BHI) medium/glycerol at -80 °C, and BHI medium/glycerol followed by RNAlater). Three nucleic acid extraction kits (QIAamp Fast DNA Stool Mini Kit, GeneJET Genomic DNA Purification Kit, and mirVana™ miRNA Isolation Kit) were evaluated to compare total nucleic acid yields. Viral DNA and RNA were extracted separately using the NucleoSpin Virus Kit, which was optimized for viral nucleic acid recovery. The DNS samples that preserved in RNAlater and extracted using the NucleoSpin Virus kit generated highest viral DNA (1.35 ± 0.12 µg) and RNA (1.57 ± 0.09 µg) (mean ± SEM) yields for metagenomics sequencing. Metagenomic sequencing and viral taxonomic assignment using Kraken2 and Kaiju, respectively showed that Kaiju allowed maximum exact matches for viral taxonomy assignment and provided high-resolution outputs for the profiling of cattle URT viromes.

**Conclusion:**

The use of an optimized nucleic acid extraction method and Kaiju-based classification enhanced viral nucleic acid recovery and taxonomic classification from low-biomass DNS samples. The findings highlighted the importance of effective storage conditions and extraction methods in maximizing viral nucleic acid yield, while demonstrating that Kaiju provided more reliable viral taxonomic assignments than Kraken2. These advancements provide a framework for more accurate assessment of the URT virome in cattle and other farm animals, enabling deeper investigation into the roles of viruses in RT health.

**Supplementary Information:**

The online version contains supplementary material available at https://doi.org/10.1186/s42523-026-00567-4.

## Introduction

The respiratory tract (RT) microbiome plays an important role in cattle health by providing colonization resistance against pathogens and influencing host susceptibility to RT infections [1]. Notably, the upper respiratory tract (URT) microbiome serves as an important reservoir for microorganisms that can colonize the lower respiratory tract (LRT) [2]. Microbial communities translocating from the URT to the LRT through aspiration and inhalation could shape microbial homeostasis and potentially affect RT health or disease progression [3, 4]. Compared to the well-studied bacterial communities in the cattle URT, research on the virome remains limited. The virome refers to the total genomes (DNA and RNA) of viruses in a microbial community, including both eukaryotic viruses that infect the host, and bacteriophages that infect resident bacteria [5]. These viruses may play a key role in modulating microbial community structure, inter-microbial competition, and immune responses in the RT [6–7]. For instance, bacteriophages can regulate the composition and functions of bacterial population [8–9], while host-targeting viruses, especially RNA viruses, may act as primary pathogens or immunomodulatory agents [10–11], triggering secondary bacterial infections and exacerbating disease progression [12].

However, virome research in cattle faces multiple challenges such as sample types, sample preservation methods, nucleic acid enrichment techniques, and a heavy reliance on high-quality nucleic acids for metagenomics sequencing [13]. Among these, sample type and preservation methods critically influence the integrity of viral nucleic acids, particularly RNA. Deep nasopharyngeal swabs (DNS) are widely used in URT microbiome studies due to their non-invasive nature and capacity to longitudinally sample microbial communities. However, the low microbial biomass and high host background pose considerable challenges for viral enrichment and nucleic acid recovery [14]. Additionally, RNA viruses are particularly vulnerable to enzymatic degradation during sample handling, transport, and storage [15], which may lead to underrepresentation or biased detection of the RNA viruses. This gap not only limits our understanding of the full spectrum of viral diversity in the bovine RT but also hampers the investigation of viral-bacterial interactions and their role in disease dynamics. Given these challenges, methodological improvements in DNS preservation and viral nucleic acid extraction are essential for accurately profiling the cattle RT virome, which directly affect downstream sequencing and taxonomic classification.

Additionally, bioinformatics pipelines significantly influence virome characterization, as different classification tools exhibit varying degrees of sensitivity and specificity. Comprehensive understanding on viral taxonomy not only depends on data processing software but also the completeness of the incorporated database. Currently, there is no well-defined pipeline for the analysis of animal symbiotic virome. To overcome these limitations, this study compared DNS sample storage methods, nucleic acid extraction protocols for both DNA and RNA, and virome taxonomy classification pipelines, aiming to refine methodologies to recover viral nucleic acids from low-biomass DNS samples and increase classification accuracy of the URT virome.

## Materials and methods

### Animal experiment and sample collection

The animal trial was conducted in accordance with the guidelines of the Canadian Council on Animal Care (CCAC) under animal care protocol (AC18-0145) approved by the Animal Care Committee at the University of Calgary. Beef calves were purchased from an auction market located in southern Alberta, Canada, and were transported to a large commercial cattle feedlot (one-time capacity of ~ 25,000 cattle) located nearby within 100 km. All experiments and sample collection were conducted over a 20-day upon arrival of heifers. A total of 24 winter-replaced heifer calves (body weight at arrival = 334.8 ± 18.1 kg per calf) with comparable physiological status and similar production background were enrolled in the study. The heifers received intranasal modified-live vaccine (against *bovine respiratory syncytial virus*, *bovine alphaherpesvirus 1*, and *bovine parainfluenza virus type 3*) and pour-on avermectin on arrival at feedlot. During the first 21 d after entering the feedlot, calves were observed by veterinarian and feedlot personnel daily for BRD symptoms. Each heifer received a reticulo-rumen temperature bolus at the enrollment to assess daily rumen temperature. Heifers with temperatures ≥ 103.5 °F were separated from the rest of the calves for additional clinical examination and sampling. Clinical examination was performed in a chute to assess heifers visually (depression, nasal and/or ocular discharge, cough, tachypnea, and dyspnea), measure rectal temperature, auscultate lungs, and perform lung ultrasound. Heifers were diagnosed as BRD, when the rectal temperature is ≥ 103.5 °F with at least one visual sign (depression, nasal and/or ocular discharge, cough, tachypnea, and dyspnea) and recorded their observations) and lung lesions at ultrasound. Following the diagnosis, the DNS samples were collected from heifers using a guarded swab with a rayon tip (27 cm; Medical Wire & Equipment, UK). After insertion into the nasal cavity, the rayon tip was extended beyond the guard and gently rubbed against the nasopharyngeal mucosa. The swab tip was then separated and transferred into a transport tube containing liquid Amies medium for subsequent processing [16]. After collection, all samples were immediately placed on ice and transported to the laboratory within 2 h and frozen at -80 °C. A total of 48 DNS samples were collected from 24 calves diagnosed with BRD (2 swabs per calf). Samples from one calf were inadequate for DNA/RNA extraction and were therefore excluded from the study.

### Optimizing DNS storage methods

**Fig. 1:**
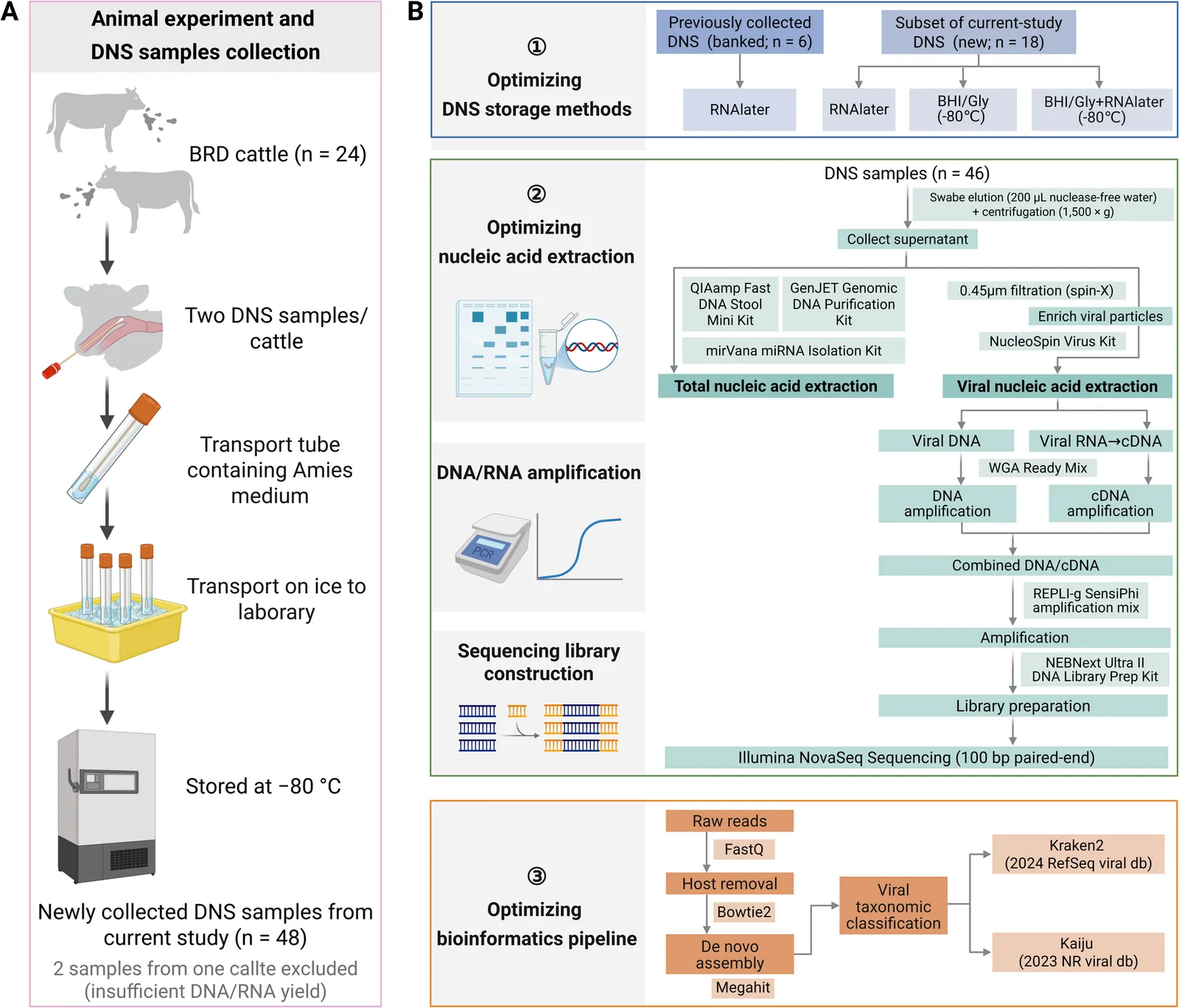
Overview of the experimental design and DNS sample processing workflow for virome profiling. (**A**) Experimental design and DNS sample collection. A total of 48 DNS samples were collected from 24 BRD cattle, with two swabs collected per animal. Samples were placed into transport tubes containing Amies medium, transported on ice to the laboratory, and stored at -80 °C. Two samples from one animal were excluded because of insufficient DNA/RNA yield. (**B**) DNS sample processing and virome profiling workflow. ① Optimization of DNS sample storage conditions. A subset of newly collected DNS samples from the current study (*n* = 18; one sample per animal to avoid biological redundancy) and previously collected banked DNS samples (*n* = 6) were used to compare preservation conditions. Newly collected samples were preserved using RNAlater, BHI/glycerol (-80 °C), or BHI/glycerol supplemented with RNAlater (-80 °C), whereas banked samples had been stored in RNAlater at -80 °C for one year. The remaining 30 newly collected DNS samples were temporarily stored as dry swabs at -80 °C for a short period (≤ 3 days) prior to processing and were subsequently preserved using the optimized storage method; these samples were not included in the storage condition comparison. ② Optimization of nucleic acid extraction and amplification protocols. Total nucleic acids were extracted using the QIAamp Fast DNA Stool Mini Kit, GenJET Genomic DNA Purification Kit, and mirVana miRNA Isolation Kit, whereas viral nucleic acids were extracted using the NucleoSpin Virus Kit after 0.45 μm filtration and viral particle enrichment. Viral RNA was reverse-transcribed to cDNA, and DNA and cDNA were subsequently amplified for library preparation and Illumina NovaSeq sequencing (2 × 100 bp). ③ Bioinformatics pipeline optimization. Raw reads were subjected to host read removal and de novo assembly, and viral taxonomic classification was performed using Kraken2 with the 2024 RefSeq viral database and Kaiju with the 2023 NR viral database. BRD, bovine respiratory disease; DNS, deep nasal swab; BHI, brain heart infusion medium; Gly, glycerol; WGA, whole genome amplification

An overview of the experimental and analytical workflow is shown in Fig. 1. Three preservation strategies were compared to identify the most suitable storage method for DNS samples. Among the 46 newly collected DNS samples obtained in the current animal experiment, 18 samples were randomly selected to evaluate storage conditions and nucleic acid extraction performance and were equally allocated to three preservation conditions (*n* = 6 per condition). In addition, six banked DNS samples, previously collected in another study (unpublished data) and stored for one year at -80 °C in RNAlater, were included to compare nucleic acid yield between newly collected and previously stored samples. Nucleic acid concentration and yield comparisons were therefore conducted using 18 newly collected samples and 6 banked samples (24 samples in total). All newly collected DNS samples (*n* = 46), including those used for storage evaluation, were subsequently included in the sequencing workflow. To avoid introducing bias prior to finalizing the optimal preservation protocol, the remaining 28 newly collected samples that were not included in the storage evaluation were immediately stored as dry swabs at -80 °C for a short period (≤ 3 days) and subsequently processed using the finalized optimal storage method. Of the 18 new DNS samples used for storage optimization, six samples were preserved in RNAlater (40 mL 0.5 M EDTA, 25 mL 1 M sodium citrate, and 700 g ammonium sulfate dissolved to a final volume of 1 L with sterile water). The remaining 12 samples were vortexed in 0.7 mL of BHI medium supplemented with glycerol; six were frozen directly at -80 °C, while the other six were supplemented with RNAlater prior to freezing.

### Optimization of nucleic acid extraction from DNS samples

Total DNA and RNA were extracted from 18 newly collected and 6 banked DNS samples using different commercial kits: the QIAamp Fast DNA Stool Mini Kit (QIAGEN, Hilden, Germany), the GeneJET Genomic DNA Purification Kit (Thermo Fisher Scientific, Waltham, MA, USA), and the mirVana™ miRNA Isolation Kit (Thermo Fisher Scientific, Waltham, MA, USA), following the manufacturers’ instructions. Each swab was vortexed in 200 µL of nuclease-free water for 30 s and centrifuged at 1,500 × g for 5 min at 4 °C. The swab tip was discarded and the supernatant was collected for downstream extraction. The collected supernatant was equally divided into three aliquots for DNA extraction using the QIAamp Fast DNA Stool Mini Kit, GeneJET Genomic DNA Purification Kit, and mirVana miRNA Isolation Kit, respectively. For viral nucleic acid extraction, both new and banked samples were processed using the NucleoSpin Virus Kit (Macherey-Nagel, Düren, Germany) following the manufacturer’s instructions. Briefly, the swab eluate was passed through a 0.45 μm filter (spin-X) and centrifuged at 19,000 × g to remove debris and enrich viral particles. A 200 µL aliquot of the filtered supernatant was transferred to a new tube containing 5 µL of proteinase K and 5.6 µg carrier RNA for downstream viral nucleic acid extraction. Subsequently, both new and banked samples were used to extract viral DNA and RNA using the NucleoSpin Virus Kit (Macherey-Nagel, Düren, Germany) following the manufacturer’s instructions. Each swab was vortexed in 200 µL of nuclease-free water for 30 s and centrifuged at 1,500 × g for 5 min at 4 °C. The supernatant was passed through a 0.45 μm filter (spin-X) and further centrifuged at 19,000 × g to remove debris and enriches viral particles. A 200 µL aliquot of the filtered supernatant was transferred to a new tube containing 5 µL of proteinase K and 5.6 µg carrier RNA, mixed thoroughly, and incubated at room temperature for 3 min. Subsequently, 200 µL of 96–100% ethanol was added, followed by vortexing and incubation for 5 min at room temperature. The entire mixture (~ 610 µL) was loaded onto the NucleoSpin^®^ Virus Column and centrifuged at 4,000 × g for 3 min. The column was washed sequentially with Buffer VW1 and Buffer VW2, each followed by centrifugation at 11,000 × g for 30 s. A final wash with VW2 was followed by centrifugation at 20,000 × g for 5 min. The column was then transferred to a new 1.5 mL elution tube and incubated at 56 °C for 5 min with the lid open. Elution was performed by adding 30 µL of RNase-free water preheated to 70 °C, followed by incubation at room temperature for 3 min and centrifugation at 20,000 × g for 3 min. The resulting eluates were stored at -80 °C until further analysis. The concentration of extracted DNA and RNA was quantified using a Qubit Fluorometer (Thermo Fisher Scientific, Waltham, MA, USA) with the Qubit dsDNA HS Assay Kit and Qubit RNA HS Assay Kit, respectively, according to the manufacturer’s instructions. Nucleic acid yield was calculated based on the measured concentration and elution volume. Samples with concentrations below the detection limit of the corresponding Qubit assay were recorded as undetectable (“Fail”) and excluded from downstream quantitative comparisons.

### Optimizing DNA/RNA amplification and sequencing library construction

Extracted viral RNA was reverse-transcribed into cDNA using the QuantiScript Reverse Transcription Mix (Qiagen, Hilden, Germany), following the manufacturer’s protocol. Briefly, 10 µL RNA was mixed with 2 µL of gDNA Wipeout buffer at 42 °C for 10 min to remove contaminating DNA. Then, 8 µL of reverse transcription mix (Table S1) was added, and the mixture was incubated at 42 °C for 60 min for cDNA synthesis, followed by enzymatic inactivation at 95 °C for 3 min. Subsequently, both DNA and cDNA were pretreated using the whole genome amplification (WGA) Ready Mix (Table S2), which included polymerase buffer, random primers, and WGA enzyme. The mixture was incubated at 42 °C for 60 min and inactivated at 95 °C for 3 min before cooling. For ligation, the pretreated nucleic acids were mixed with a ligation buffer system (Table S3), followed by incubation at 24 °C for 30 min and heat inactivation at 95 °C for 5 min. Finally, ligated products were amplified using the REPLI-g SensiPhi amplification system (Table S4), which employs Φ29 DNA polymerase for high-fidelity isothermal amplification. The amplified products were used for downstream library preparation. Given the low-biomass nature of DNS samples, multiple negative controls (NCs) were included throughout the experimental workflow and sequenced in the same run. These comprised a library preparation negative control (NC-library), whole transcriptome amplification negative control (NC-wta), whole genome amplification negative control (NC-wga), nucleic acid extraction blanks processed with (NC-with-carries) and without (NC-no-carries) carrier nucleic acids, and an environmental bench swab control (NC-bench_swab). All NCs were processed using the same extraction, amplification, library preparation, sequencing, and bioinformatic analysis pipeline as the cattle DNS samples.

Metagenomics sequencing libraries were prepared using NEBNext^®^ Ultra II DNA Library Prep Kit (New England BioLabs Inc., MA), which is compatible with low-input samples (as low as 500 pg). Paired-end (2 × 100 bp) sequencing was performed on an Illumina NovaSeq platform (Illumina Inc., San Diego, CA, USA). To minimize background contamination, filtering was performed at the taxonomic feature level following taxonomic assignment of assembled contigs. Taxa detected in ≥ 2 extraction- or library-related NCs were classified as likely contaminants. Such taxa were removed from downstream diversity and community analyses if present at low abundance in biological samples (< 10 reads per sample and prevalence < 5%). Prevalence was defined as the proportion of biological samples in which a taxon was detected. Taxa detected exclusively in the environmental bench swab control were annotated as potential environmental background signals and were not automatically excluded unless meeting the contaminant criteria described above.

### Bioinformatics pipeline and taxonomic classification

Raw paired-end reads were quality-filtered and adapter-trimmed using Trimmomatic (v0.39) in paired-end mode, with Illumina adapter removal, sliding-window quality trimming (SLIDINGWINDOW:4:15), and a minimum read length cutoff of 75 bp. Read quality before and after trimming was assessed using FastQC (v0.12.1) [17]. Host-derived reads were removed by aligning quality-filtered reads to the bovine reference genome (UMD3.1) using Bowtie2 (v2.5.4) in end-to-end, very-sensitive mode [18]. Host-filtered paired-end reads were assembled *de novo* using MEGAHIT (v1.2.9) [19], with a minimum contig length cutoff of 500 bp. Viral contigs were taxonomically classified using two independent classifiers to assess the consistency of taxonomic assignments. Kraken2 (v2.1.6) was applied using the RefSeq viral database (release 2024), downloaded in September 2024 and built following the standard Kraken2 database construction workflow (including k-mer indexing). Kaiju (v1.10.1) was run in Greedy mode against the NCBI non-redundant (NR) protein database (release 2023-03-10), using the following parameters: minimum match length = 11, minimum match score = 75, allowed mismatches = 2, and maximum E-value = 0.01. Viral genome types were annotated based on taxonomic assignments following the International Committee on Taxonomy of Viruses (ICTV) taxonomy framework (https://ictv.global/taxonomy).

Viral relative abundance was estimated by mapping quality-filtered, host-removed reads back to viral contigs using Bowtie2 (v2.5.4). Only properly paired and uniquely mapped reads were retained. To account for differences in viral genome length and sequencing depth across samples, read counts were normalized using reads per kilobase per million mapped reads (RPKM). Relative abundance was calculated by dividing the RPKM value of each viral contig by the sum of RPKM values within each sample.

### Statistical analysis

Statistical comparisons of nucleic acid yield and concentration across preservation conditions, extraction kits, and sample types were conducted using nonparametric tests due to the relatively small sample size (*n* = 6 per preservation condition). Each DNS sample originated from a different calf, and no repeated measurements were obtained from the same animal across preservation treatments; therefore, animal identity could not be included as a random effect in the statistical model. For comparisons involving three preservation conditions, the Kruskal-Wallis test was applied followed by Dunn’s multiple comparisons test with Bonferroni correction. For two-group comparisons (banked vs. new samples), the Mann-Whitney U test was used. All analyses were performed using SPSS Statistics (v22.0; IBM). Data are presented as mean ± SEM. Statistical significance was defined as *P* < 0.05, and a trend was considered at 0.05 ≤ *P* < 0.1.

## Results and discussions

### Optimization of DNS samples storage methods and nucleic acid extraction from low-biomass DNS samples

**Fig. 2:**
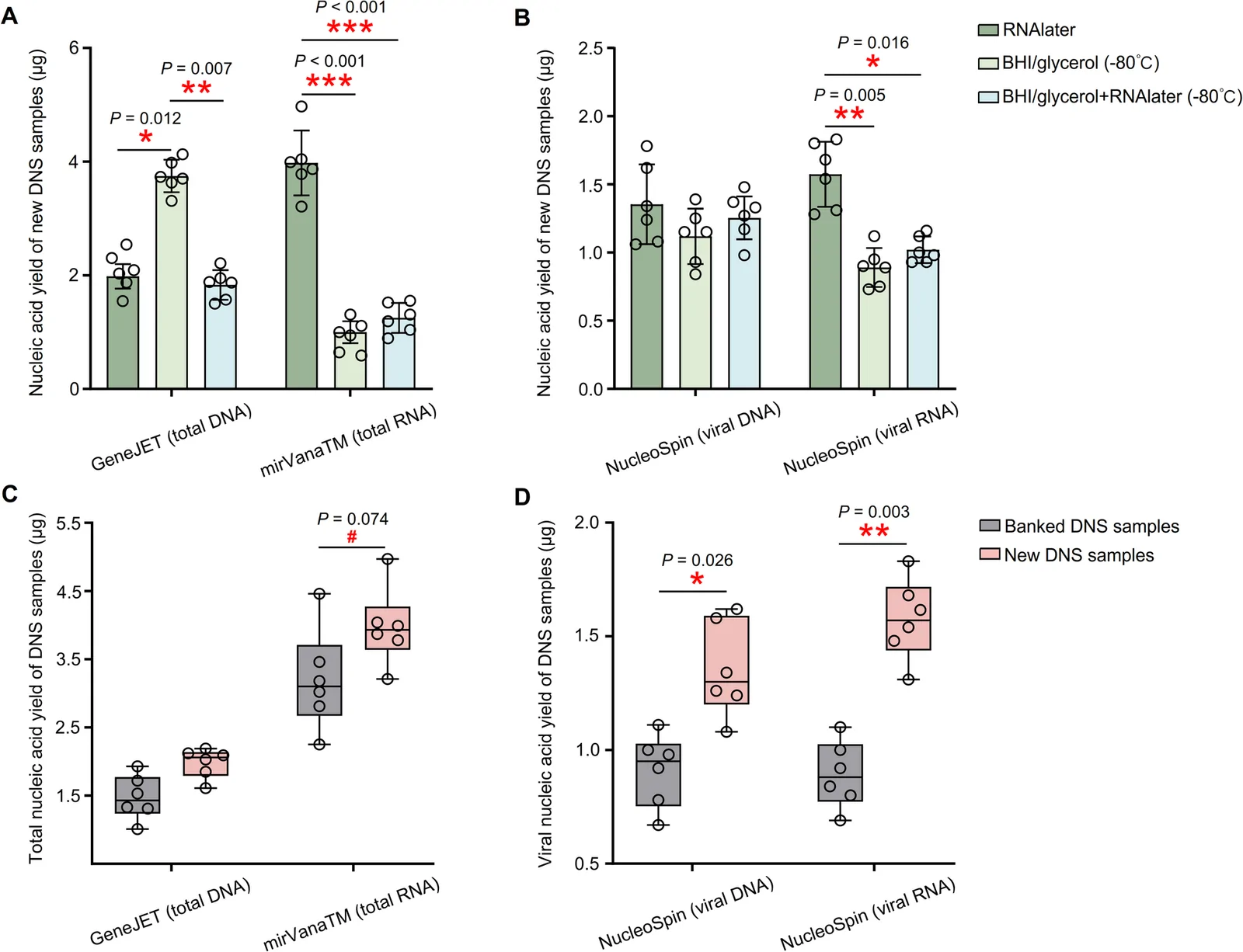
Optimization of nucleic acid preservation and extraction for DNS samples collected from the cattle URT. (**A**, **B**) Total DNA, RNA, and viral nucleic acid yields (µg) of new DNS samples preserved using RNAlater, BHI/glycerol (-80 °C), or BHI/glycerol with RNAlater (-80 °C), and extracted using GeneJET (total DNA), mirVana™ (total RNA), or NucleoSpin Virus Kit (viral DNA/RNA). (**C**) Comparison of total nucleic acid yield between new and banked DNS samples using GeneJET and mirVana™ kits. (**D**) Qubit-based quantification of viral DNA and RNA concentrations (ng/µL) in new versus banked DNS samples extracted with the NucleoSpin Virus kit. Bars or boxplots represent mean ± SEM (*n* = 6). ***P < 0.001, **P < 0.01, *P < 0.05, #0.05 ≤ *P* < 0.1 (trend). URT, upper respiratory tract; BHI, brain heart infusion. Panels **A**-**B** and **C**-**D** are displayed separately due to the different ranges of nucleic acid yields between total nucleic acids and viral nucleic acids, requiring different y-axis ranges for clear visualization

When evaluated for total DNA extraction, the new DNS samples preserved in brain heart infusion (BHI) medium/glycerol at -80 °C and extracted using GeneJET genomic DNA Purification kit generated higher total DNA (mean ± SEM) (3.75 ± 0.12 µg) yield compared to those stored in RNAlater (1.98 ± 0.09 µg; *P* = 0.012) or BHI medium/glycerol + RNAlater (1.83 ± 0.11 µg; *P* = 0.007) (Fig. 2A). The QIAmp Fast DNA Stool Mini Kit failed to extract detectable DNA from several DNS samples (Table S5), with multiple yields falling below the Qubit detection limit. For total RNA extraction, new DNS samples preserved in RNAlater and extracted using mirVana™ miRNA isolation kit achieved higher RNA yield (3.98 ± 0.23 µg) than those stored in BHI medium/glycerol at -80 °C (1.00 ± 0.08 µg; *P* < 0.001) or BHI medium/glycerol + RNAlater (1.25 ± 0.11 µg; *P* < 0.001) (Table 1; Fig. 2A). Viral nucleic acids were extracted using the NucleoSpin Virus Kit, and among the newly collected DNS samples, viral DNA yield did not differ significantly among preservation conditions (Table S6). Similarly, the viral RNA yields were higher in the RNAlater group (1.57 ± 0.09 µg) compared to those stored in BHI/glycerol (0.89 ± 0.06 µg; *P* = 0.005) and BHI/glycerol + RNAlater (1.02 ± 0.04 µg; *P* = 0.016) (Table S6; Fig. 2B). Additionally, total RNA yield in new samples (3.98 ± 0.23 µg) tended to be higher than those from banked samples (stored DNS samples from 1y ago) (3.20 ± 0.30 µg; *P* = 0.074) (Fig. 2C). Both viral DNA (1.35 ± 0.12 µg) and viral RNA (1.57 ± 0.09 µg) in new DNS samples were higher than those in banked samples (viral DNA: 0.91 ± 0.07 µg, *P* = 0.026; viral RNA: 0.89 ± 0.06 µg, *P* = 0.003) (Fig. 2D).

**Table 1:** Viral taxonomy assignment using Kraken2 and Kaiju with updated databases

Items	Kraken2 (2024 RefSeq viral database)	Kaiju (2023 NR viral database)
No. of viral contigs	23,931	42,977
No. of singleton removal	23,748	42,871
No. of viral taxa (singleton removal)		
Kingdom	6	6
Phylum	13	7
Class	18	19
Order	20	27
Family	36	37
Genus	122	88
Species	191	182
No. of viral species by nucleic acid type		
dsDNA viruses	131	95
ssDNA viruses	9	9
dsRNA viruses	0	1
ssRNA viruses	37	70
Unclassified viruses	14	7

These findings showed the key influence of both preservation strategy and extraction protocol on nucleic acid recovery from low-biomass DNS samples, particularly when targeting different nucleic acid types (total and viral DNA/RNA). Preservation buffers differ in terms of chemical composition, preservation mechanisms, and suitability for specific nucleic acids. BHI medium/glycerol is traditionally used for cryopreservation of viable bacteria, where glycerol serves as a cryoprotectant to mitigate freezing-induced cell lysis [20]. This makes it particularly effective in maintaining intact microbial cells, thereby facilitating total DNA extraction, especially when paired with the GeneJET Genomic DNA Purification kit, which relies on mechanical lysis and silica-membrane binding for recovering high-molecular-weight genomic DNA [21, 22]. However, due to the lack of an RNA-stabilizing agent, BHI medium/glycerol provided no protection against enzymatic RNA degradation, resulting in poor recovery of both total and viral RNA. In contrast, RNAlater rapidly permeates cells/tissues and irreversibly inactivates RNases [23, 24], making it widely used for stabilizing and preserving labile RNA in field-collected samples. Meanwhile, RNAlater has been shown to preserve the infectivity of both enveloped and non-enveloped viruses [23], indicating its capacity to stabilize free or weakly protected viral nucleic acid during the early stages of sample handling. RNAlater-preserved samples yielded higher levels of total RNA and viral RNA/DNA in the current study, indicating its ability to better preserve viral nucleic acids in DNS samples. However, as the new and banked samples originated from different animals and were not matched pairs in this study, the observed differences may reflect both storage-related effects and inter-animal variability. Therefore, these comparisons should be interpreted with caution when considering long-term storage effects. Future studies using paired designs would help better control for host-related variability.

Although RNAlater stabilizes total RNA and viral nucleic acids, its fixative properties may interfere with cell lysis and protein digestion, reducing the efficiency of total DNA extraction from host and bacterial cells [25–27]. However, viral DNA encapsulated in small viral particles is less impacted [28], which may account for the higher recovery of viral DNA compared to total DNA in RNAlater-preserved samples. The mirVana™ miRNA Isolation kit employs a phenol: chloroform lysis step followed by selective RNA binding on a glass-fiber filter. This kit is optimized to efficiently capture both large and small RNAs, explaining its robust performance on low-biomass respiratory swabs [29]. Although the QIAamp Fast DNA Stool Mini Kit is widely used for extracting microbial and host genomic DNA from high-biomass fecal samples [30], its application to swab-based RT samples, which often contain only limited quantities of microbial and viral nucleic acids, may therefore be problematic. The kit is formulated with aggressive lysis conditions, including strong detergents and mechanical disruption, which are optimized for dense, resilient microbial communities. However, when applied to low-input samples, such as DNS, these harsh conditions may lead to excessive shearing or loss of nucleic acids [31]. Additionally, limited starting material and incompatibility with low-yield matrices may further contribute to the reduced recovery or extraction failure observed in this study.

The NucleoSpin Virus Kit exhibited consistent high efficiency in extracting both viral DNA and RNA. The chaotropic salt-based lysis buffer and silica membrane columns of this kit are optimized for free nucleic acids and viral particles, making it suitable for virome studies where viral genomes are fragmented, encapsulated, or present in extremely low abundance [32, 33]. RNAlater again outperformed other preservation methods when paired with this kit, yielding the highest amounts of both viral DNA and RNA. This suggests that the combination of RNase inactivation and optimized viral lysis chemistry is essential to enhance the recovery of viral nucleic acids prior to sequencing for virome profiling, especially when targeting RNA viruses. To our knowledge, the combination of BHI/glycerol and RNAlater has not been previously reported. In this study, we tested this hybrid approach with the intent to combine the cryoprotective effect of BHI/glycerol and the RNA-stabilizing properties of RNAlater. However, this combination failed to achieve additive benefits. Instead, it produced lowest yield across all extraction methods compared to each individual buffer. This likely reflects certain incompatibilities: BHI/glycerol is designed to maintain microbial viability [20, 34], whereas RNAlater acts as a fixative [35]; their opposing purposes and physicochemical differences, such as altered ionic strength or viscosity, may disrupt cell integrity or interfere with nucleic acid purification. Moreover, the high-salt environment of RNAlater may compromise the cryoprotective function of glycerol [36]. The high viscosity and hygroscopic nature of glycerol may hinder the effective removal of RNAlater prior to nucleic acid purification [37], particularly for silica column-based kits, which are sensitive to buffer composition and sample complexity. These findings suggest that mixing preservation buffers with different purposes reduces nucleic acid recovery.

### Optimizing bioinformatics pipeline to profile cattle URT virome

**Fig. 3:**
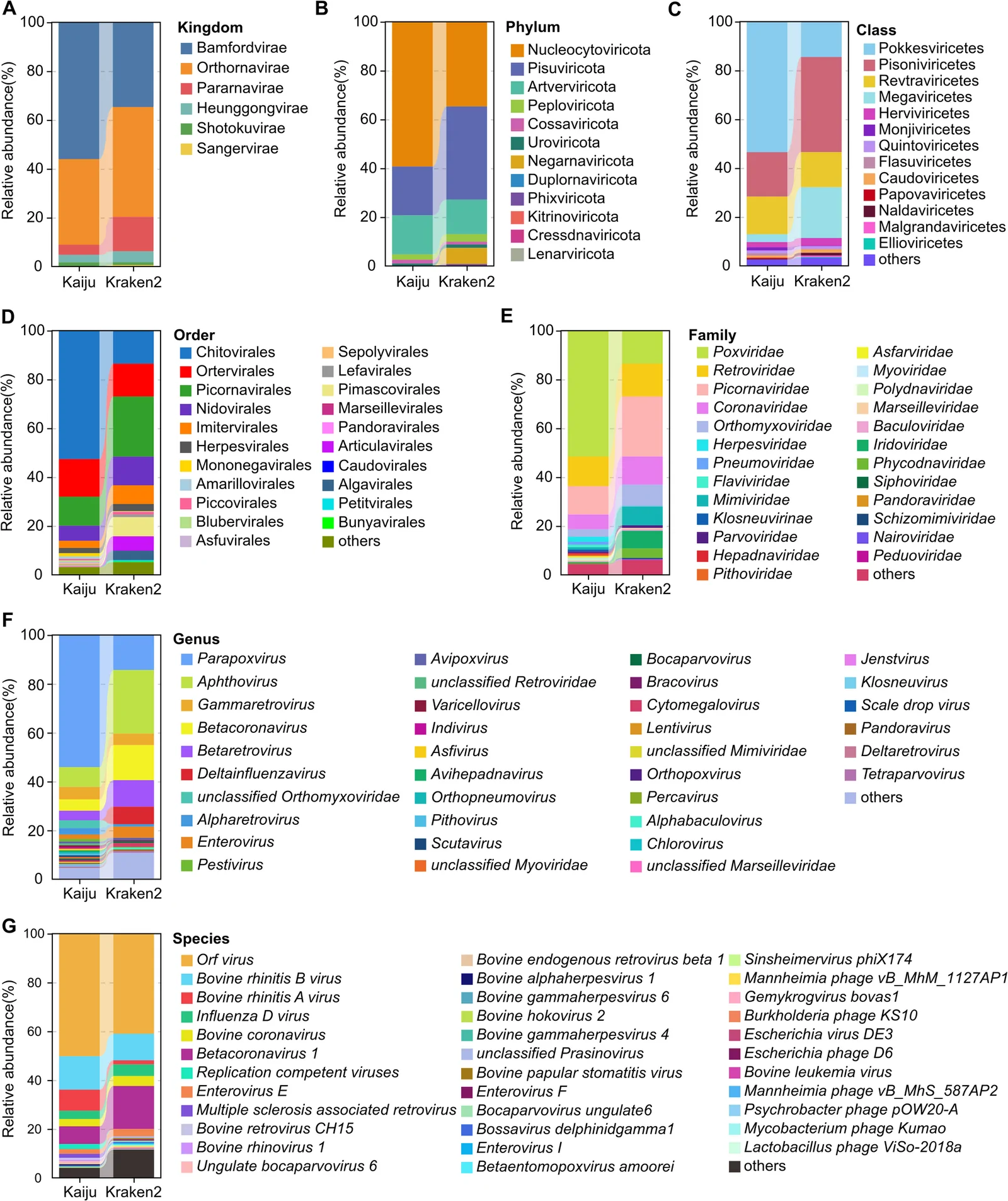
Comparison of taxonomic assignments of the bovine URT virome generated by Kaiju and Kraken2. Stacked bar charts show the mean relative abundance (%) of viral taxa across 46 DNS samples at each taxonomic rank: (**A**) Kingdom, (**B**) Phylum, (**C**) Class, (**D**) Order, (**E**) Family, (**F**) Genus, and (**G**) Species. Taxonomic profiles were generated using Kaiju (2023 NR database) and Kraken2 (2024 RefSeq viral database). Relative abundances were calculated based on genome-length-normalized read counts (RPKM) and expressed as percentages within each sample. Only taxa with relative abundance ≥ 0.1% in at least one classifier are shown, remaining taxa are grouped as “others.” URT, upper respiratory tract; DNS, deep nasopharyngeal swab

To evaluate taxonomic classification strategies for cattle URT virome, we compared commonly used pipelines for general metagenomic analysis, with a focus on their performance in classifying viral contigs derived from DNS samples. Although the mirVana and GeneJET kits yielded high amounts of total RNA or DNA, they were not used for virome sequencing in this study. Our goal was to evaluate how preservation conditions influence nucleic acid recovery, and to assess virome profiling performance specifically using an optimized viral extraction and sequencing workflow. Therefore, only nucleic acids extracted using the NucleoSpin Virus Kit were used for downstream virome sequencing. After quality control and host read removal, an average of 19,595,099 ± 772,532 (mean ± SEM) reads per sample were retained from the initial 24,963,460 ± 1,074,683 (mean ± SEM) raw reads (Table S7). A total of 23,931 and 42,977 viral contigs were annotated by Kraken2 and Kaiju, respectively. After singleton removal, 23,748 contigs remained in the Kraken2 dataset and 42,871 in the Kaiju dataset (Table 1). At the taxonomic level, Kraken2 assigned the viral contigs to 6 kingdoms, 13 phyla, 18 classes, 20 orders, 36 families, 122 genera, and 191 species (Table 1; Table S8). In comparison, Kaiju assigned the viral contigs to 6 kingdoms, 7 phyla, 8 classes, 27 orders, 37 families, 88 genera, and 182 species (Table 1; Table S9). At species level, Kraken2 identified *Orf virus* (41.04%) and *Betacoronavirus 1* (17.63%) as the most abundant species, followed by *Bovine rhinitis B virus* (BRBV, 10.88%), *influenza D virus* (IDV, 4.75%), and *Bovine rhinitis A virus* (BRAV, 1.64%) (Fig. 3). Kaiju similarly identified *Orf virus* (50.88%) as the dominant species, but additionally detected BRBV (13.71%), BRAV (8.64%), IDV (3.39%), *Bovine coronavirus* (BCoV) (3.02%), *Betacoronavirus 1* (7.24%), *Bovine rhinovirus 1* (BRV1) (1.64%), *Ungulate bocaparvovirus* 6 (1.64%) and *Bovine alphaherpesvirus 1* (1.58%), many of which were absent or below detection in Kraken2 results (Fig. 3). Although algorithmic and database differences, the above viral species annotated by both Kraken2 and Kaiju are known to be associated with cattle RT-associated viruses [6, 38, 39].

**Fig. 4:**
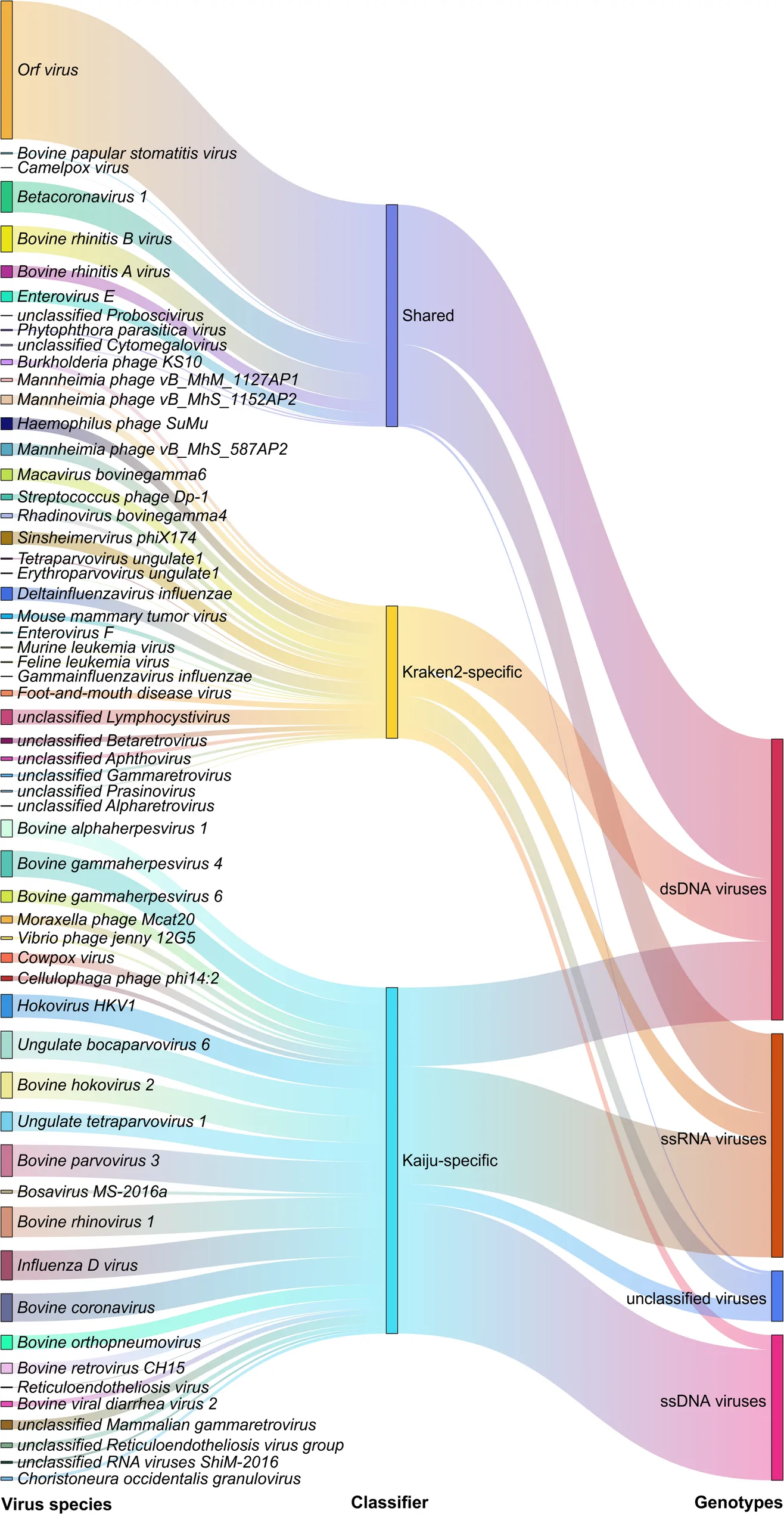
Genome-type classification of viral species identified by Kraken2 and Kaiju. Viral species were categorized as shared (detected by both Kraken2 and Kaiju), Kraken2-specific, or Kaiju-specific. Flows link virus species to classifier and genome type (dsDNA, ssDNA, ssRNA, or unclassified). Link thickness represents mean relative abundance (%) across samples

Based on established genome-type classifications, viral contigs were grouped into double-strand (ds) DNA, single-strand (ss) DNA, dsRNA, and ssRNA viruses. Kraken2 identified 131 dsDNA viruses, 9 ssDNA viruses, and 37 ssRNA viruses, and 14 unclassified viruses, with no dsRNA viruses detected (Table 1). In contrast, Kaiju detected 95 dsDNA viruses, 9 ssDNA viruses, 1 dsRNA virus, 7 unclassified viruses, and 70 ssRNA viruses (Table 1). To further assess genome-type differences between classifiers, viral species were grouped by detection category and genome type (Fig. 4). Shared detections were primarily composed of dsDNA and ssRNA viruses, with *Orf* virus representing the dominant dsDNA component and *Betacoronavirus 1* and *bovine rhinitis* viruses contributing substantially to the ssRNA fraction. Kraken2-specific detections were largely enriched in dsDNA bacteriophages, including representative taxa such as *Mannheimia* and *Streptococcus* phages, whereas Kaiju-specific detections included a broader range of host-associated ssRNA and ssDNA viruses, such as Bovine orthopneumovirus, *Bovine viral diarrhea virus 2*, and *ungulate bocaparvoviruses*. Overall, these patterns indicate genome-type-dependent differences in detection capacity between the two pipelines.

One critical concern in low-biomass virome studies is the potential amplification bias introduced during the WGA step. WGA, particularly multiple displacement amplification-based approaches, may preferentially amplify certain genome types (e.g., small circular ssDNA viruses), generate uneven genome coverage, or introduce quantitative distortions in relative abundance estimates [40]. Although WGA is commonly applied to enhance nucleic acid yield in low biomass samples, it may affect the representation of viral community structure [41]. In the present study, WGA was applied uniformly across all samples to ensure consistency in processing. Therefore, although absolute abundance estimates should be interpreted with caution, comparative patterns across preservation methods and sample types remain informative. Future studies considering amplification-free library preparation or spike-in controls may improve quantitative accuracy. In addition, given the low-biomass nature of DNS samples, contamination represents an inherent concern in virome studies. In the present study, multiple NCs were included throughout the extraction, amplification, and library preparation processes, and contaminant filtering was performed at the taxonomic feature level following classification. Respiratory viruses reported in the main dataset were not detected in NC libraries at levels comparable to those observed in biological samples. Although low-level background contamination cannot be entirely excluded, the consistent detection of key high-abundance respiratory viruses across samples and classifiers supports the robustness of the primary virome patterns observed.

The observed differences in viral classification between Kraken2 and Kaiju likely reflect fundamental distinctions in their algorithms and reference databases. Kraken2 applies a fast, k-mer-based exact matching strategy against the RefSeq viral database, enabling efficient classification of well-characterized viral sequences with high nucleotide conservation [42]. However, classification accuracy in Kraken2 is highly dependent on database completeness, its sensitivity may be limited when analyzing divergent or fragmented viral sequences, as reads lacking close reference matches are often left unclassified or assigned only to higher taxonomic levels [43]. This limitation may partly account for Kraken2’s lower detection of cattle RT-associated viruses, especially RNA viruses, as many of them exhibit high sequence variability and incomplete representation in existing databases. In contrast, Kaiju employs protein-level alignment against the more expansive and taxonomically diverse NCBI NR database [44, 45], which includes a broader spectrum of viral taxa, such as environmental, uncultured, and evolutionarily divergent viruses. This protein-space strategy increases tolerance to synonymous substitutions and frameshift mutations, thereby improving Kaiju’s ability to detect viral homologs with low nucleotide identity [44–46]. This may also explain the fewer unclassified viruses in our dataset reported by Kaiju (*n* = 7) compared to Kraken2 (*n* = 14). In a benchmarking study [46] using real clinical respiratory samples, Kaiju consistently outperformed other classifiers by demonstrating higher sensitivity in pathogen detection, greater classification accuracy (as measured by the area under the ROC curve), and stronger correlation between read abundance and PCR Ct-values, particularly for RNA viruses with high sequence variability. These features enable Kaiju to achieve improved classification performance, especially when analyzing reads from viral genera that are underrepresented in reference databases. This advantage likely underpins Kaiju’s broader recovery of cattle RT-associated viruses observed in the current study, especially RNA viruses, which are predominant in bovine respiratory disease (BRD) [6, 39]. Notably, Kaiju uniquely identified *Bovine orthopneumovirus*, BRV1, *Bovine retrovirus CH15*, and BVDV-2, which are all well-documented contributors to the BRD complex [6, 38, 39] but were not detected by Kraken2. Although Kraken2 did capture key high-abundance viruses such as *Orf virus* and *Betacoronavirus 1*, as well as some phages targeting common respiratory bacteria, its underrepresentation of important RNA viruses suggests limitations in classifying complex respiratory viromes using nucleotide-level matching alone. These findings suggest that future studies targeting complex and underrepresented viromes, such as those associated with the cattle RT, particularly RNA viruses, may consider using protein-level classifiers like Kaiju to improve taxonomic resolution. In addition, integrating multiple classification strategies and expanding reference databases with a broader representation of livestock-related viruses could further enhance the accuracy and comprehensiveness of viral detection and annotation. Collectively, the current study revealed that Kaiju out-perform other bioinformatics pipelines in profiling URT virome, particularly in its capacity to detect a broader range of host-associated RNA and DNA viruses. Its protein-level alignment strategy appears to be effective in capturing evolutionarily diverse and low-abundant viral taxa. However, since Kraken2 detected bacteriophages critical for regulating the URT microbiota that Kaiju missed, combining the two tools may yield a more complete characterization of the virome, capturing both host viruses and phage signals.

It is noticeable that viral detection was largely limited to DNA viruses in earlier metagenomic studies of the cattle RT [47, 48]. Although some recent studies have begun to identify RNA viruses [6, 39, 49], their consistent recovery, especially from low-biomass RT samples, remains technically challenging, likely due to the difficulties in preserving and obtaining high quantity and quality of RNA [13, 15]. The current study significantly improved the recovery of RNA viruses and expanded the capacity to characterize both DNA and RNA viruses in DNS samples simultaneously. Importantly, the RNA viruses identified in this study, such as BCoV, IDV, BRV1, and BVDV-2 are known viruses causing BRD [12, 50–52]. Hence, the optimized workflow to study virome in low-biomass RT samples could support future investigations into host-bacteria-virome interactions and their impact on bovine respiratory health.

## Conclusions

We designed an optimized workflow from sample preservation to bioinformatic analysis for profiling the URT virome using DNS. The use of RNAlater for sample preservation and the NucleoSpin Virus kit for nucleic acid extraction provided high-quality materials suitable for metagenomic library preparation. Kaiju detected a broader range of viral taxa, particularly RNA viruses, likely reflecting its protein-level alignment strategy and database composition, whereas Kraken2 provided complementary detection of bacteriophages through k-mer-based classification. These differences emphasize methodological complementarity rather than inherent biological superiority of one classifier over the other. This optimized workflow facilitates comprehensive and biologically informative URT virome profiling while acknowledging the influence of database composition and classification strategy on taxonomic outputs. However, the current study did not evaluate the potential impact of different swab types (i.e., rayon-tipped, cotton-tipped, flocked swabs, or even cytobrushes to collect more epithelial cells) on virome composition, which remains an important consideration for future standardization.

## Supplementary Information

Below is the link to the electronic supplementary material.

Supplementary Material 1

## Data Availability

The sequence data have been submitted to the NCBI sequence reads archive (SRA) under accession number PRJNA1259072.

## Abbreviations

RT: Respiratory tract
URT: Upper respiratory tract
LRT: Lower respiratory tract
DNS: Deep nasopharyngeal swabs
BRD: Bovine respiratory disease
BHI: Brain heart infusion
ds: Double-strand
ss: Single-strand
WGA: Whole genome amplification
